# Isolation and Characterization of Antagonistic Bacteria* Paenibacillus jamilae* HS-26 and Their Effects on Plant Growth

**DOI:** 10.1155/2019/3638926

**Published:** 2019-03-27

**Authors:** Xiaohui Wang, Qian Li, Junkang Sui, Jiamiao Zhang, Zhaoyang Liu, Jianfeng Du, Ruiping Xu, Yanyan Zhou, Xunli Liu

**Affiliations:** ^1^College of Life Science, Shandong Agriculture University, No. 61, Daizong Street, Taian, Shandong 271018, China; ^2^College of Forestry, Shandong Agriculture University, No. 61, Daizong Street, Taian, Shandong 271018, China

## Abstract

Soilborne pathogens affect plant growth and food production worldwide. The application of chemical fertilizers and pesticides to control plant diseases has harmful effects; fortunately, plant growth-promoting rhizobacteria can be used as a potential alternative strategy. Here,* Paenibacillus jamilae* HS-26 was selected for its highly antagonistic activity against several soilborne pathogens. The bacterium synthesized hydrolytic enzymes and released extracellular antifungal metabolites and volatile organic compounds—primarily, N, N-diethyl-1, 4-phenylenediamine, which was detected by gas chromatography-mass spectrometry and shown to inhibit fungal mycelial growth. Furthermore, HS-26 was useful for nitrogen fixation, phosphate and potassium solubilization, and siderophore and indoleacetic acid production.* In vitro* tests and pot experiments revealed that HS-26 considerably increased plant biometric parameters. Illumina MiSeq sequencing data showed a significant reduction in soilborne pathogens and increase in beneficial bacteria in the wheat rhizosphere after treatment with strain HS-26.

## 1. Introduction

Plant diseases critically endanger agricultural resources. In particular, soilborne pathogens cause dramatic yield and economic losses, with fungi being the most aggressive pathogens [1, 2]. In the past few decades, chemical fungicides have had a critical role in controlling plant diseases and increasing crop yield. Until now, suppression of soilborne pathogens mainly relied on chemical pesticides. However, recently, scientists have reported that long-term use of chemical agents can cause adverse effects, including environmental contamination, resistant-plant pathogen outbreak, progressively greater production costs owing to the over-expenditure on these chemicals, and even toxicity in humans [3]. Fortunately, biological control, using plant growth-promoting rhizobacteria (PGPR) as biocontrol agents (BCAs) that interfere with plant pathogens, could be an alternative to chemical control measures and could avoid the problems caused by chemical methods for plant protection [4].

Biological control using antifungal rhizobacteria to suppress plant diseases has been extensively investigated. Rhizosphere soil of healthy plants is considered an excellent source of PGPR [5], which stimulates plant growth through various mechanisms [6].* Paenibacillus polymyxa* strain SQR-21 and* Paenibacillus pasadenensis* strain R16 are primarily known for their ability to prevent plant diseases [2, 7]. The action of* Bacillus amyloliquefaciens* subsp. Plantarum S499 relies on its potential to produce multiple antimicrobial metabolites against phytopathogens and its ability to induce systemic resistance in plants [8].* Pseudomonas saponiphila* strain YW aids the growth of pepper seedlings via its ability to synthesize indole acetic acid (IAA) and solubilize phosphate [3]. However, it should be noted that the size of introduced bacterial populations positively correlates with increased disease control [9]. The ability to colonize the rhizosphere has been considered a critical prerequisite for the success of PGPR [10]. To be effective, PGPR must establish and maintain a sufficient population size within the rhizosphere [11]. Hence, the development of indigenous biocontrol strains that suit local environments may help improve the competitiveness with* in situ* microorganisms and effectiveness at suppressing local pathogen strains [12].

Despite the increasing number of scientific papers dealing with biological control, an insufficient number of products are available in the market [13]. It is known that the efficiency of mycelial growth inhibition* in vitro* does not always correlate with biocontrol efficacy under natural conditions [14, 15]. Furthermore, the production of antifungal metabolites or other components is subject to complex regulation by an array of environmental factors, and these metabolites may not be equally expressed under* in vitro* and natural soil conditions [16, 17]. Hence, understanding the mechanisms of disease control may help to control soilborne diseases efficiently.

The present study aimed to (1) isolate and screen BCAs as potential inoculants with application within the same area from which they were isolated; (2) determine the direct and indirect plant growth-promoting properties of the BCAs* in vitro*; (3) test the BCAs for their effects on seed germination and seedling growth; (4) reveal the impact of the BCAs on wheat growth and microbial communities in the rhizosphere of wheat.

## 2. Materials and Methods

### 2.1. Collection of Soil Samples and Bacterial Isolation

Cucumber rhizosphere soil samples were collected from a vegetable planting base located in Tai'an City, Shandong Province, China. Cucumber seedlings at the beginning of flowering were uprooted with intact roots, and the excess bulk soil was removed by gentle shaking. Soil adhering to the roots was considered rhizosphere soil [18], which was collected from roots by dipping and gentle shaking in sterile distilled water under aseptic conditions, after which the samples were mixed on a table concentrator for 30 min. The resulting solutions were serially diluted up to 10^−6^, inoculated on potato dextrose agar (PDA) plates, and incubated at 28°C ± 2°C for 24 h [19]. Colonies were selected from these plates and maintained using PDA medium at 4°C. All selected strains were stored at −80°C in Luria-Bertani broth (LB) containing 30% glycerol for further study.

### 2.2. Screening and Identification of Antagonistic Bacteria

The antagonistic abilities of the isolates were determined in dual-plate confrontation assays against the plant pathogens* Fusarium oxysporum*,* Botryosphaeria ribis*,* Bipolaris sorokiniana*,* Botryosphaeria dothidea*,* Alternaria* (Nees),* Fusarium pseudograminearum, Colletotrichum gloeosporioides*, and* Rhizoctonia solani. *After 4 days, the diameters of the zone of fungal growth inhibition around the bacteria were measured and recorded. Then, the bacteria with the most potent inhibitory activity were selected and identified by 16S rRNA gene sequence, which was amplified using universal primers 27F and 1492R [20]. The obtained PCR products were sequenced, and the nucleotide sequences were compared with the GenBank database using BLAST (https://blast.ncbi.nlm.nih.gov/Blast.cgi).

### 2.3. Mechanisms of Fungal Inhibition

The ability to produce extracellular antifungal metabolites and volatile organic compounds (VOCs) and the antagonistic effect on the radial growth of plant pathogens* F. oxysporum*,* B. sorokiniana*, and* R. solani* were tested in a dual-plate confrontation assay [21]. Furthermore, the type and the relative content of VOCs produced by antagonistic bacteria were measured and analyzed by gas chromatography-mass spectrometry (GC-MS); the detailed method was mentioned in our previous article [22]. In the present study, the most prominent and unreported volatile components were selected, and their effects on the plant pathogens* F. oxysporum*,* B. ribis*,* B. sorokiniana*,* B. dothidea*,* R. solani*, and* F. pseudograminearum* were examined. The centers of PDA plates were inoculated with 5-mm discs of pregrown phytopathogenic fungi, and a piece of filter paper (1 cm × 1 cm) moistened with 10 ml of distilled water was placed on another plate. A concentration gradient of the volatile commercial compound was applied to the filter paper quickly [23]. All half-plates were placed face to face and sealed to prevent VOC loss. Distilled water was used as a control. After 3 days of incubation at 30°C, the diameter of the phytopathogenic fungal colonies was measured and recorded. Additionally, extracellular hydrolytic enzymes including chitinase, protease, cellulose, and glucanase, which participate in the mechanisms underlying fungal growth inhibition, were measured qualitatively and quantitatively as previously described [22].

### 2.4. Growth-Promoting Characteristics of Isolates

In addition to the antifungal activity, PGP activities of antagonistic bacteria were analyzed. Siderophore production was detected using the chrome azurol S (CAS) method [24, 25]. Indole-3-acetic acid (IAA) production was measured based on the chromogenic reaction of Salkowski reagent with IAA [26]. Phosphate solubilization was determined using the molybdenum blue method [3]. Potassium-solubilization efficiency was estimated by flame photometry [27]. The nitrogen-fixing ability was evaluated using the method described previously [28]. All experiments were performed in triplicate, and the results were expressed as mean values and standard deviations.

### 2.5. HS-26 Effects on Cucumber Seed Germination and Plant Growth

The germination of cultivated cucumber seeds (Zhongnong No.8) inoculated with HS-26 cells was assessed. Strain HS-26 was grown overnight in LB broth at 30°C at 200 r/min, and the bacterial suspension was centrifuged at 1073 ×* g* for 10 min. The pellet was resuspended in sterile ddH_2_O and adjusted to 4 × 10^8^ CFU/mL. The seed surface was sterilized with 75% ethanol for 1 min, followed by 1% sodium hypochlorite for 30 min, after which the seeds were washed extensively with sterile water [29]. Cucumber seeds were then inoculated with HS-26 strain suspension for 30 min at 28°C, while the control seeds were treated with an equal amount of sterile water [6]. A total of 200 seeds, treated and untreated, were seeded onto platters (20 cm × 30 cm), containing filter paper moistened with sterilized distilled water, for the seed germination assay. After 1 week, the rate of germination of cucumber seeds was evaluated. In addition, 50 cucumber seeds (25 treated/25 untreated) were sown onto each platter in the same manner. After 15 d, the seedlings were harvested to determine the effects of HS-26 treatment on plant growth, and fresh/dry weight and length of the shoots and roots were measured. All experiments were performed at room temperature (20 ± 2°C) and in triplicate. Results were expressed as mean values and standard deviations.

### 2.6. Pot Experiment

#### 2.6.1. Pot Experiment Design

A pot experiment was conducted to evaluate the effect of strain HS-26 on the growth of wheat seedlings. The experimental design was a completely randomized block design with three replicates for each treatment and 10 pots per replicate. The experiment included the following 2 treatments: a noninoculated control and HS-26 strain-inoculated treatment. Wheat seeds were surface-sterilized as described above. HS-26 strain was grown in LB broth at 30°C on a shaking incubator at 200 rpm for 48 h. Subsequently, HS-26 strain was centrifuged at 1073 ×* g* for 10 min; the pellet was resuspended in sterile ddH_2_O and adjusted to 6–8 × 10^8^ CFU/mL. Five sterilized wheat seeds were sown in 10 L clay tile pots containing soil collected from a local field under wheat cultivation. For the HS-26 strain treatment, the roots of 1-week-old plantlets were inoculated with 20 mL diluted culture suspension of HS-26 strain; the roots of the control plantlets were inoculated with the same volume of sterile water. All the pots were irrigated once before sowing to ensure proper seed germination and then regularly watered during crop growth as per agronomic practices. After 2 months, the wheat seedlings were uprooted separately to determine plant biomass indices. The rhizosphere soil was then carefully collected from 10 random soil cores from pots, and the samples were pooled to yield one composite sample per replicate. The soil samples were passed through a 2-mm sieve, thoroughly homogenized, and stored at −80°C for the analysis of microbial community structure.

#### 2.6.2. DNA Extraction, PCR Amplification, and Illumina MiSeq Sequencing

To reveal whether and how the HS-26 strain positively influenced the microbial community structure, soil genomic DNA from control and HS-26-treated samples was extracted using the Soil DNA kit (Omega Bio-Tek, China). The universal bacterial primers 515F and 907R were used to amplify the V4–V5 regions of the bacterial 16S rRNA gene and the fungal-specific primers ITS1F and 2043R to amplify the fungal ITS1 region. Then, amplicons were purified and pooled in equimolar amounts and paired-end sequenced (2 × 250) on an Illumina MiSeq platform (Illumina, USA) by Majorbio Bio-pharm Technology Co., Ltd. (Shanghai, China).

#### 2.6.3. Processing of Illumina MiSeq Sequencing Data

After removing the barcode and primer sequences, the raw FASTQ files were demultiplexed and quality-filtered using QIIME (version 1.9.1 (http://qiime.sourceforge.net/)), and the chimeric sequences were removed using UCHIME [30]; operational taxonomic units (OTUs) were clustered with 97% similarity cut-off using UPARSE (version 7.1; http://drive5.com/uparse/); alpha diversity index was calculated using Mothur (http://www.mothur.org/); the 16S RNA reads were assigned to bacterial taxonomic groups using RDP classifier (http://rdp.cme.msu.edu/) against the Silva (SSU123) 16S rRNA database using a confidence threshold of 70%; the taxonomy of each ITS rDNA gene sequence was analyzed by RDP classifier against the UNITE 7.0/ITS database using a confidence threshold of 70%; raw Illumina sequencing data from the current study were submitted to the NCBI Sequence Read Archive (SRA) under the accession number SRP132621.

### 2.7. Statistical Analysis

All experiments were performed in triplicate, and all statistical analyses were performed using SAS version 8.0 software (SAS Institute, Inc.). Differences in mean values were considered significant when* P* < 0.05.

## 3. Results

### 3.1. Isolation and Identification of Strain HS-26

**Figure 1:**
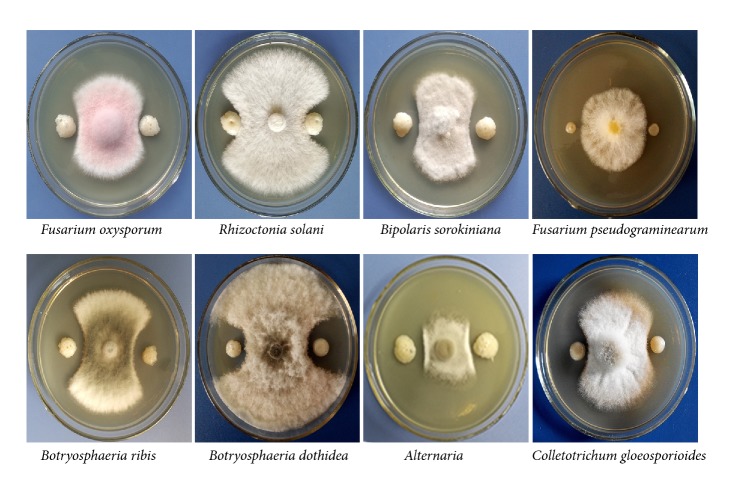
*In vitro* antagonism of strain HS-26 against phytopathogens. Antagonism test between strain HS-26 and plant pathogens by dual-culture assays on PDA medium 4 days after incubation.

In the present study, 127 bacterial strains, obtained from the rhizosphere soil of healthy cucumber seedlings at the beginning of flowering, were evaluated using* in vitro* antagonism assays. Strain HS-26 was selected for further analyses due to its potent and broad-spectrum antagonistic activity as shown in Figure 1; the diameter of the zones of fungal growth inhibition was determined and recorded in Table 1. Subsequently, strain HS-26 was identified as* P. jamilae* based on the phylogenetic analysis of the 16S rRNA sequence, which has been deposited at the National Center for Biotechnology Information (NCBI) under the accession number MH211597.

### 3.2. Fungal Inhibition Mechanisms

#### 3.2.1. Inhibition of Mycelial Growth by Extracellular Antifungal Metabolites and VOCs

**Figure 2:**
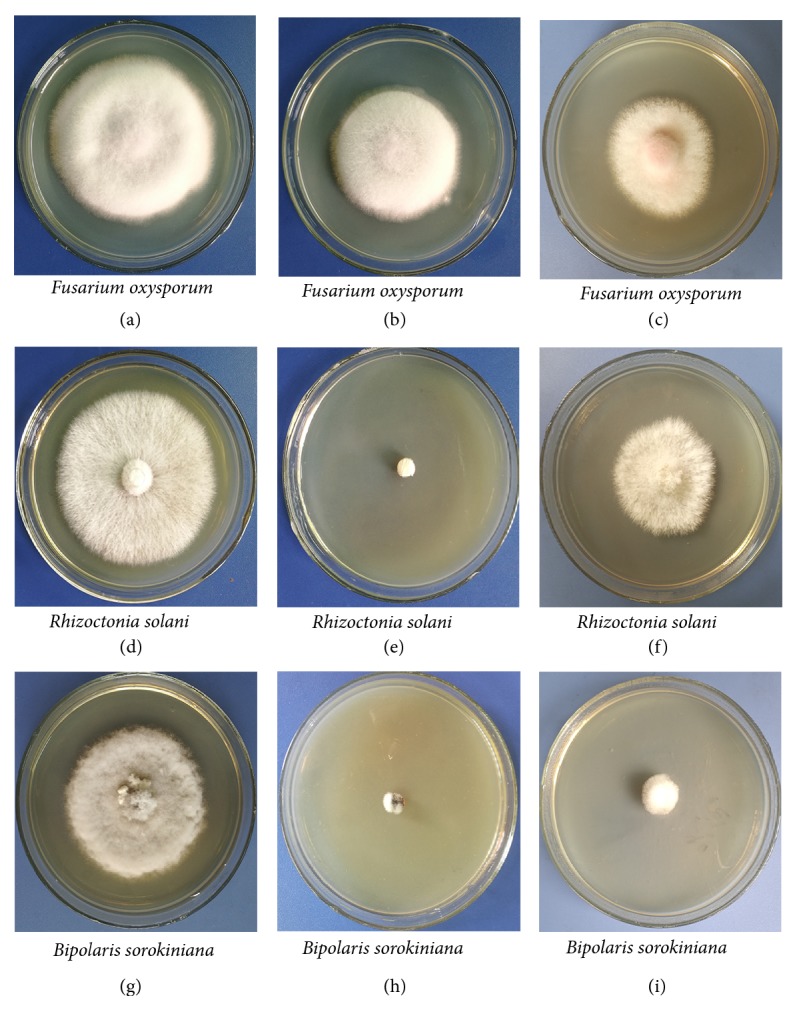
Effect of the extracellular antifungal metabolite and VOC production by HS-26 strains on radial growth of plant pathogens. As compared to control (left), phytopathogen mycelial plug growth was inhibited by the extracellular antifungal metabolite (middle) and VOC production by HS-26 strains (right).

Compared with controls, radial growth of all tested fungal pathogens was expectedly inhibited by extracellular antifungal metabolites (Figure 2). The growth of* B. sorokiniana* and* R. solani* was completely inhibited, while that of* F. oxysporum* was reduced by 17.65%. Similar to the extracellular antifungal metabolites, the VOCs produced by strain HS-26 also reduced the mycelial growth, with the inhibition of* F. oxysporum*,* B. sorokiniana*, and* R. solani* being approximately 46.30%, 63.86%, and 44%, respectively.

#### 3.2.2. GC-MS Analysis and Antifungal Activity of VOCs

**Figure 3:**
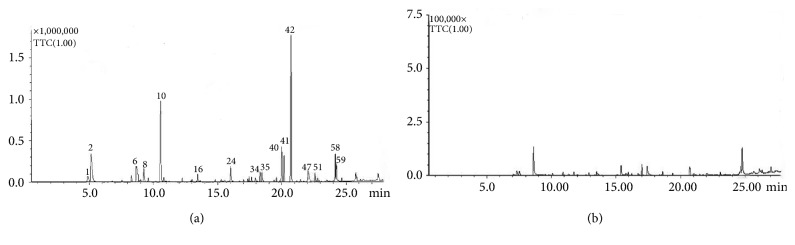
Identification and analysis of VOCs produced by HS-26 cells on PDA medium by GC-MS. VOCs produced by HS-26 cells (a) and control PDA medium (b). Chromatogram corresponding to volatile organic compounds produced by HS-26: ethanol (1), acetone (2), acetoin (6), 2-methyl-1-butanol (8), 2,3-butanediol (10), 2-methylenecyclohexanol (16), trans,trans-3,5-heptadien-2-one (24), 2-nonanone (34), 2-nonanol (35), 2-undecanone,6,10-dimethyl- (40), 2-tridecanol (41), N,N-diethyl-1, 4-phenylenediamine (42), undecanone (47), 2-(2-methylpropyl)-3-(1-methylethyl)pyrazine (51), 2-dodecanone (58), 2-hexadecanol (59).

**Figure 4:**
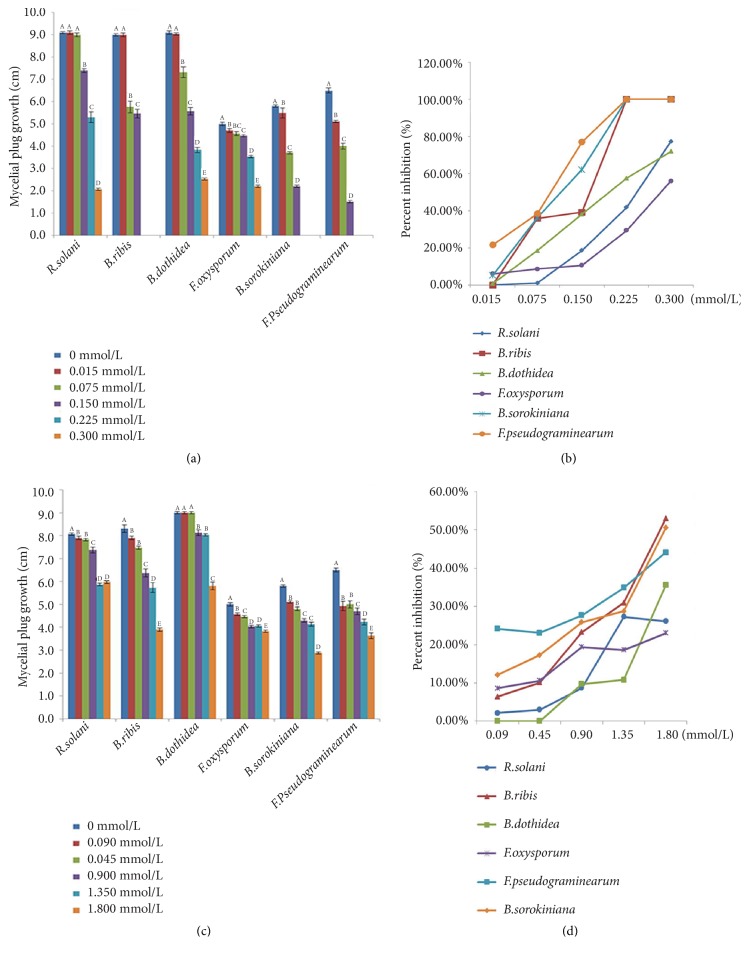
Inhibitory activities of the identified volatile organic compounds (VOCs) against plant pathogens. The diameter of the pathogen colony (a) and percent of growth inhibition (b) of* F. oxysporum*,* B. ribis*,* B. sorokiniana*,* B. dothidea*,* Alternaria* (Nees), and* R. solani* exposed to different concentrations of 2-methyl-1-butanol. The diameter of the pathogen colony (c) and percent of inhibition (d) of these plant pathogens exposed to different concentrations of N, N-diethyl-1, 4-phenylenediamine. Bars indicate standard errors (n = 3). Different letters above columns indicate significantly different results (*P* < 0.05, Student's t-test).

The VOCs produced by strain HS-26 were analyzed by GC-MS, and 69 compounds were detected (Figure 3). The compounds with a relative peak area of more than 1% are listed in Table 2, with N, N-diethyl-1, 4-phenylenediamine, 2, 3-butanediol, and acetone being the top three dominant VOCs. Among them, a few compounds have been previously reported, such as acetoin and 2, 3-butanediol, which can promote the growth of* Arabidopsis thaliana* [33]; acetone and 2-undecanone, which showed strong nematicidal activities [32]; ethanol, which manifested the highest antibacterial activity against* Staphylococcus aureus* and* Streptococcus pyogenes* [31]; and 2-methylenecyclohexanol, 2-nonanone, 2-nonanol, 2-(2-methylpropyl)-3-(1-methylethyl)pyrazine, and 2-dodecanone, which exhibited strong inhibition of mycelial growth and spore germination [34, 35]. In the present study, we examined the effect of 2-methyl-1-butanol and N, N-diethyl-1, 4-phenylenediamine on the mycelial growth of* F. oxysporum*,* B. ribis*,* B. sorokiniana*,* B. dothidea*,* R. solani*, and* F. pseudograminearum*. 2-Methyl-1-butanol was purchased from Aladdin Industrial Corporation (Shanghai, China), and N, N-diethyl-1, 4-phenylenediamine was acquired from Macklin Reagent Company (Shanghai, China). As shown in Figure 4, 2-methyl-1-butanol completely inhibited the growth of* F. pseudograminearum, B. ribis*, and* B. sorokiniana* at a concentration of 0.225 mmol/mL* in vitro*. N, N-diethyl-1, 4-phenylenediamine did not show pathogen-killing ability at the maximum concentration of 1.8 mmol/L, which was set in the present study; however, it exerted distinct inhibitory effects on mycelial growth and the diameter of the formed colonies. The rate of inhibition was positively correlated with the concentration of N, N-diethyl-1, 4-phenylenediamine.

#### 3.2.3. Fungal Cell Wall-Degrading Enzymes

**Figure 5:**
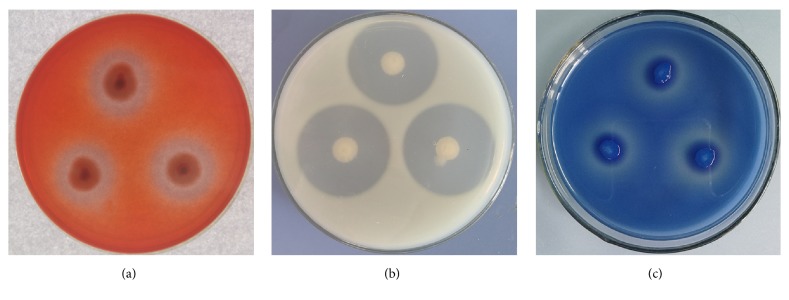
Fungal cell wall-degrading enzymes produced by HS-26 cells. (a) Cellulase activity determined using carboxyl methyl cellulose (CMC) agar plates; (b) Protease activity determined using skim milk agar plates; (c) Glucanase activity determined using Pachyman solid medium supplemented with 6% aniline blue.

The production of fungal cell wall-degrading enzymes by strain HS-26 was studied both qualitatively and quantitatively. HS-26 cells were inoculated onto carboxyl methyl cellulose agar plates, skim milk agar plates, and pachyman solid medium supplemented with 6% aniline blue and then all plates were incubated at 30°C. After 3 days, semicircular hyaline zones around bacterial colonies were formed on the specific agar media (Figures 5(a), 5(b), and 5(c)), demonstrating fungal cell wall-degrading enzyme production by HS-26 cells. Quantification of the produced enzymes showed that, after 3 days, cellulase, glucanase, and protease levels reached 62.76 ± 1.35 U/mL, 4.13 ± 0.53 U/mL, and 15.56 U/mL, respectively. It was interesting to note that the level of HS-26 strain-secreted chitinase was up to 1012.67 ± 65.36 U/mL, although no semicircular hyaline zone was formed on the plate containing colloidal chitin.

### 3.3. Evaluation of PGP Abilities

In addition to the antifungal activity, strain HS-26 also showed positive results for nitrogen fixing (6.95 ± 0.05 mg/g), phosphate solubilization (38.93 ± 5.09 *μ*g/L), potassium dissolving (21 ± 1.32 *μ*g/mL), and siderophore (4.67 ± 0.86 *μ*g/mL) and IAA (4.83 ± 0.14 *μ*g/mL) production tests.

### 3.4. In Vivo Seed Germination and Growth-Promoting Effects

HS-26 strain positively affected the germination of cultivated cucumber seeds (Figure S1). Following the coating of seeds with HS-26 cells, the percentage of germination increased to 75%, while the germination rate in the control group was only 60%. After 15 days, growth parameters of the HS-26-treated seedlings were superior to those of the untreated controls (Table S1). Root length, shoot height, fresh biomass, and dry biomass increased by 19.22%, 30.53%, 11.72%, and 4.17%, respectively. In addition, the cucumber seedlings treated with these cells had more lateral root.

### 3.5. Pot Experiment

#### 3.5.1. Growth-Promoting Effect of Bacterial Treatment on Wheat

For the* in vivo* pot experiment, wheat was chosen to test the active contribution of the HS-26 strain to plant growth. Significant effects on plant growth were observed for the plants treated with strain HS-26 compared with those for the nontreated control (Table 3). The predominant influences observed were an enhancement of the wheat seedling dry biomass (by 26.67%), fresh biomass (by 38.89%), and the root/shoot ratio (by 31.45%). Although there was no significant difference in wheat height between the HS-26 treatment and control groups, we found that the wheat seedlings treated with HS-26 strain had a higher number of lateral roots, which enabled them to obtain sufficient water and nutrients from the soil.

#### 3.5.2. Illumina MiSeq Sequencing and Sequence Analysis

More than 30,000 fungal and 20,000 valid bacterial reads were obtained for each replicate through a sequence optimization process, and these sequences were grouped into 1347 fungal OTUs and 4345 bacterial OTUs using a 3% dissimilarity cut-off. Rarefaction and Shannon curves tended to approach the saturation plateau with the increase in sequencing number (Figure S2), which indicated that sequencing capability was sufficiently large to completely capture the diversity of these communities. The *α*-diversities of the soil microbial communities are presented in Table 4; there were no significant differences in bacterial community richness indices (ACE and Chao) and diversity indices (Shannon and Simpson) between the HS-26 treatment and control groups. However, for the fungal community, the lower ACE and Chao values after HS-26 treatment demonstrate that the HS-26 strain treatment group maintained a lower richness than the control group, and the lower Shannon values in the HS-26 treatment group indicated that the HS-26 strain treatment group maintained a lower diversity than the control group. Simpson values showed a similar trend for the fungal community.

#### 3.5.3. Taxonomic Composition Analysis

**Figure 6:**
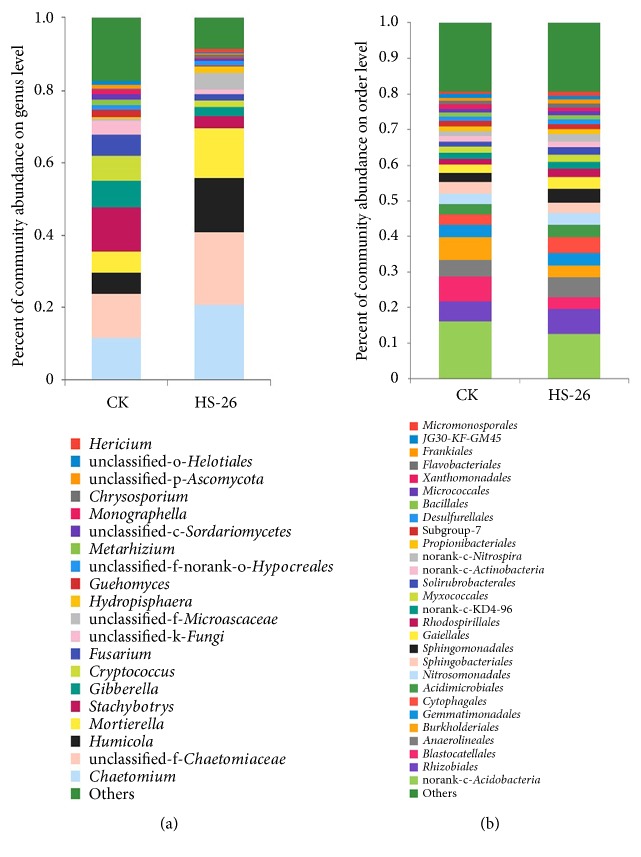
The relative abundance (%) of all fungi on the genus level (a) and all bacteria (b) on the order level in the rhizosphere soil of HS-26 and control treatments.

**Figure 7:**
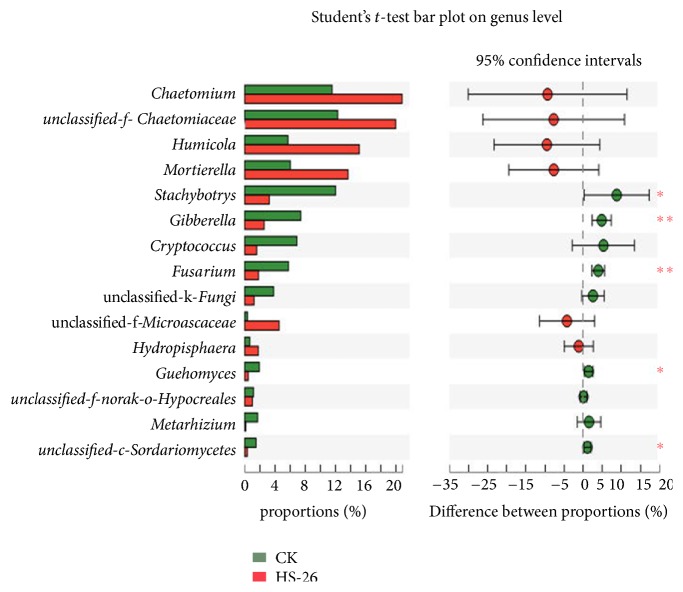
Multipathogenic fungal genera difference test between HS-26 treatment and control treatments. Error bars indicate standard errors (n = 3). Columns with asterisks are significantly different (*P* < 0.05) according to Student's t-test.

All the sequences were classified from the phylum level down to the species level using the Mothur program. For the fungal community, three known fungal phyla were detected, with Ascomycota being the most dominant phylum and accounting for 91.36% and 76.88% in the HS-26 treatment and control groups, respectively (Figure S3). At the genus level, thirteen known fungal genera were detected (Figure 6(a)). Among them, the relative contents of* Chaetomium*,* Humicola*, and* Mortierell*a in the HS-26 treatment group were higher than those in the control group, while* Stachybotrys*,* Gibberella*, and* Fusarium* were significantly lower in the HS-26 treatment group (Figure 7). Furthermore, after mining useful information hidden in the original data, the relative abundances of* Monographella*,* Bipolaris*,* Volutella*, and* Idriella* in the HS-26 treatment group were found to be decreased as compared to those in the control group (Table S2). For the bacterial community, ten bacterial phyla were detected both in the HS-26 treatment and control groups, with Proteobacteria, Acidobacteria, Actinobacteria, and Chloroflexi ranking as the top four bacterial phyla (Figure S3). At the order level, the relative contents of plant-associated beneficial bacteria, such as Micromonosporales, Rhizobiales, Acidimicrobiales, Frankiales, Gemmatimonadales, Sphingomonadales, Nitrosomonadales, Rhodospirillales, Desulfurellales, and Bacillales, were higher in the HS-26 treatment than in the control groups. Conversely, potential pathogens including Burkholderiales and Xanthomonadales were reduced in the HS-26 treatment group (Figure 6(b))

## 4. Discussion

Members of the genera* Alternaria* [36],* Botrytis* [37],* Fusarium* [38], and* Rhizoctonia* [39] are pathogens that infect a wide range of plants, including several crops, vegetables, and fruits, leading to dramatic economic losses. Conversely, bacteria associated with plant roots are potential agents for biological control of soilborne plant pathogens and for promoting plant growth [40]. An appropriate* in vitro* assessment system is necessary to select and isolate beneficial indigenous strains. In the present study, we implemented an integrated approach [12] to test isolates for a variety of plant growth-promoting characteristics. In our study, strain HS-26 exhibited more than one plant growth-promoting trait and is expected to be beneficial for seedling growth under multiple types of adverse conditions.

*P. polymyxa* is widely known as a source of plant growth-promoting [41] and biocontrol agents [42]. However, reports on the application of other* Paenibacillus *spp. in biocontrol are few [2]. In the present study, the strain* P. jamilae* HS-26 was selected for its efficient antagonistic activity. The reason may be the ability of strain HS-26 to produce antibacterial metabolites and hydrolases, which directly act on the cell wall when in contact with the fungi and hence prevent normal radial growth [16, 43, 44]. Furthermore, VOCs produced by beneficial bacteria that contribute to inhibiting plant pathogen growth and spore germination have also received considerable attention [23, 33, 38, 45, 46]. As compared with diffusion antibiotics, VOCs can spread over a long distance, and fungistatic microenvironments expand around the antagonist communities [34]. Therefore, microbial antagonist strains capable of producing volatile compounds with potent inhibitory activity against plant pathogens are more likely to prevent pathogenic fungi from infecting plants, kill surviving spores in the soil, and limit both the production and the establishment of the disease [40]. In our study, the VOCs produced by strain HS-26 were analyzed by GC-MS, and 69 compounds were detected (Table 2). Antifungal activity assays demonstrated that the main components 2-methyl-1-butanol and N, N-diethyl-1, 4-phenylenediamine distinctly inhibited the growth of fungal mycelia (Figure 4). To the best of our knowledge, this is the first report of the inhibitory effects of 2-methyl-1-butanol and N, N-diethyl-1, 4-phenylenediamine on fungal mycelial growth.

Furthermore, strain HS-26 also exhibited other growth-promoting abilities. In this study, cucumber seeds inoculated with HS-26 strain had higher germination rates than the control; this may be due to the synthesis of hormones such as IAA, which can trigger the activity of specific enzymes that promote early germination and increase plumule and radicle length [47]. In a pot experiment, the increased wheat dry weight and fresh weight implied that the HS-26 strain could colonize the root system of wheat and play a role in promoting growth. Interestingly, there was a large difference in the ratio of weight increase caused by strain HS-26 between dry weight (27%) and fresh weight (39%). A possible explanation for this is that the increase in fresh weight was related to water absorption, while the increase in dry weight was related to organic matter accumulation. As indicated in the Results section, both wheat and cucumber seedlings treated with HS-26 strain had a higher number of lateral roots, which enabled them to obtain sufficient water and nutrients from the soil. However, the accumulation of organic matter is a slow process, and organic matter accumulation is less in the early stage of plant growth. This accounted for the large difference in the ratio of weight increase between dry weight and fresh weight.

Illumina MiSeq sequencing analysis demonstrated that inoculation with strain HS-26 reduced the diversity and richness of the soil fungal communities. The relative abundances of potential pathogens, including* Gibberella*,* Fusarium*,* Monographella*,* Bipolaris*,* Volutella*, and* Idriella*, were reduced as compared with those of the control. Among them,* Gibberella* and* Fusarium* are the main pathogens of Fusarium head blight (FHB), which is a devastating worldwide disease of wheat, barley, and other small grain cereals [48]. For the bacterial community, richness indices (ACE and Chao) and diversity indices (Shannon) showed no significant change after treatment with strain HS-26; however, at the order level, the relative abundances of well-known beneficial bacteria, such as Acidimicrobiales, Rhodospirillales, Cytophagales, Sphingomonadales, Nitrosomonadales, Gemmatimonadales, Desulfurellales, Bacillales, Rhizobiales, Frankiales, and Micromonosporale, were higher in the strain HS-26 treatment group than in the control group. Among them, most species of Bacillales are reported as biological control agents against many fungal and bacterial pathogens [49]. Rhizobiales, Sphingomonadales, Rhodospirillales, Nitrosomonadales, and Frankiales participate in the nitrogen cycle [50, 51], while Gemmatimonadales, Acidimicrobiales, and Micromonosporale have the potential to improve soil fertility and plant growth efficiency [52, 53]. Conversely, the relative contents of potential plant pathogens Burkholderiales and Xanthomonadales were higher in control groups. Most species of Xanthomonadales and Burkholderiales are pathogens of plant diseases, such as black rot of crucifers and rice bacterial blight, caused by* Xanthomonas campestris* pv.* campestris *and* Xanthomonas oryzae*, respectively [54, 55]; bacterial wilt caused by* Burkholderia plantarii*; and banana bacterial wilt caused by* Burkholderia solanacearum* [56, 57]. Overall, from the above results, we discovered that strain HS-26 had direct antagonistic effects against plant pathogens and allowed beneficial microbes to accumulate more easily in the wheat rhizosphere.

## 5. Conclusion

Plant growth promotion and biocontrol activities are the main requirements for commercial microbial agents used in sustainable agriculture [58]. Our study indicated that strain HS-26 introduced into the wheat rhizosphere might play essential roles in the transition of soil from a suppressive to favorable condition. Hence, we suggest that strain HS-26 has the potential to be used in sustainable agriculture. Although our isolates need further testing under field conditions, we are confident that our findings can be transferred to the field, because the strains were isolated from the same environment in which they are intended to be used. To the best of our knowledge, this is the first report on screening* P. jamilae *as potential BCAs, and it comprehensively elaborates the mechanisms of biocontrol of soilborne diseases.

## Figures and Tables

**Table 1:** Antibiotic capacity of *Paenibacillus polymyxa *strain HS-26 against plant pathogenic fungi *in vitro*.

Fungal isolates	Antagonistic effect (zone of inhibition diameter [mm])
*Fusarium oxysporum*	1.1 ± 0.0
*Botryosphaeria ribis*	6.2 ± 0.0
*Bipolaris sorokiniana*	3.9 ± 0.2
*Botryosphaeria dothidea*	3.1 ± 0.3
*Rhizoctonia solani*	0 ± 0
*Colletotrichum gloeosporioides*	4.2 ± 0.1
*Alternaria *(Nees)	3.0 ± 0.1
*Fusarium pseudograminearum*	Ineffective

Notes: values are means ± SD. “Ineffective” indicates that the HS-26 strain has no ability to inhibit the growth of phytopathogens; “0” indicates that the HS-26 strain has the ability to inhibit the growth of phytopathogens to some extent; however, it failed to form an obvious zone of inhibition.

**Table 2:** Relative volatile organic compound (VOC) levels produced by HS-26 cells.

Serial number	Rt (min)	Area (%)	Components	PGP trait	Reference
1	4.879	1.78	Ethanol	Antagonism	[31]
2	5.143	8.22	Acetone	Nematicidal activities	[32]
6	8.658	5.67	Acetoin	Growth-promoting	[33]
8	9.244	2.35	2-Methyl-1-butanol		Not reported
10	10.564	12.91	2,3-Butanediol	Growth-promoting	[33]
16	13.444	1	2-Methylenecyclohexanol	Antagonism	[34]
24	16.008	2.39	trans,trans-3,5-Heptadien-2-one		Not reported
34	18.3	1.2	2-Nonanone	Antagonism	[35]
35	18.453	1.31	2-Nonanol	Antagonism	Patent (CN201510866778.5)
40	19.989	4.75	2-Undecanone,6,10-dimethyl		Not reported
41	20.17	3.12	2-Tridecanol	Negative for antagonism	Patent (CN201510866778.5)
42	20.713	26.78	N,N-Diethyl-1,4-phenylenediamine		Not reported
47	22.053	1.83	Undecanone	Nematicidal activities	[32]
51	22.598	1.12	2-(2-Methylpropyl)-3-(1-methylethyl)pyrazine	Antagonism	[34]
58	24.159	3.03	2-Dodecanone	Antagonism	Patent (CN201510866778.5)
59	24.251	2.01	2-Hexadecanol	Negative for antagonism	Patent (CN201510866778.5)

**Table 3:** Effect of the HS-26 strain on growth parameters of wheat seedling.

Treatment	Shoot height	Root length	Dry weight	Fresh weight	Root/shoot
	(cm)	(cm)	(g)	(g)	ratio
CK	40.76 ± 0.83a	17.78 ± 1.16a	0.30± 0.04b	1.26 ± 0.16b	0.1809 ± 0.011b
HS-26	40.53 ± 40.09a	19.44 ± 0.70a	0.38 ± 0.02a	1.75± 0.06a	0.2378 ± 0.011a

Notes: values are means ± SD (n = 30). Means sharing a common letter within the same column are not significantly different at *P* < 0.05. “HS-26” denotes wheat seedlings treated with a suspension of strain HS-26 cells. “CK” denotes wheat seedlings treated with an equal volume of sterile water.

**Table 4:** Diversity and richness indices of bacterial and fungal community from HS-26 treatment and control.

Index	Bacteria		Fungi	
	CK	HS-26	CK	HS-26
Shannon	6.67 ± 0.13a	6.63 ± 0.10a	3.40 ± 0.12a	2.56 ± 0.08b
Simpson	0.0032 ± 0.00a	0.0034 ± 0.00a	0.07 ± 0.02b	0.15 ± 0.02a
Ace	2084.66 ± 62.60a	2028.66 ± 78.74a	307.55 ± 7.47a	240.51 ± 13.63b
Chao	2107.38 ± 47.71a	2040.40 ± 89.44a	304.18 ± 12.46a	238.15 ± 9.06b
Coverage	0.9810	0.9813	0.9963	0.9989

Notes: Chao and ACE values are indicators of species richness. Shannon and Simpson values are indicators of species diversity. Values are means ± SD (n = 3). Means sharing a common letter within the same column are not significantly different at* P* < 0.05.

## Data Availability

All data generated or analyzed during this study are included in this published article.

## Abbreviations

BCAs: Biocontrol agents
CFU: Colony-forming units
GC-MS: Gas chromatography-mass spectrometry
IAA: Indole acetic acid
ITS: Internal transcribed spacer
LB: Luria-Bertani
OTUs: Operational taxonomic units
PDA: Potato dextrose agar
PGPR: Plant growth-promoting rhizobacteria
VOCs: Volatile organic compounds.

## Supplementary Materials

Supplementary Materials Figure S1: Effects of HS-26 on cucumber seed germination and growth. Seeds germination assay. (a) Seeds coated with HS-26 strain cells; (b) seeds uncoated with HS-26 strain cells as blank control; (c) growth promotion of cucumber seedlings by* Paenibacillus jamilae* HS-26 compared with the untreated control. Figure S2: bacterial and fungal rarefaction curves and Shannon curves depicting the effect of strain HS-26 and control treatments on the number of OTUs. (a) Rarefaction curves of bacteria from HS-26 and control treatments; (b) Shannon curves of bacteria from HS-26 and control treatments; (c) rarefaction curves of fungi from HS-26 and control treatments; (d) Shannon curves of fungi from HS-26 and control treatments. Figure S3: the relative abundance (%) of all bacteria and fungi on the phylum level in the rhizosphere soil of HS-26 and control treatments. (a) The relative abundance of all detected bacterial phyla in control group; (b) the relative abundance of all detected bacterial phyla in HS-26 treatment; (c) the relative abundance of all detected fungal phyla in control group; (d) the relative abundance of all detected fungal phyla in HS-26 treatment. Table S1: HS-26 effects on the germination rate and growth of cucumber. Table S2: percent of pathogenic fungi abundance in HS-26 treatment and control groups.

## References

[1] Gal-Hemed I., Atanasova L., Komon-Zelazowska M., Druzhinina I. S., Viterbo A., Yarden O. (2011). Marine isolates of trichoderma spp. as potential halotolerant agents of biological control for arid-zone agriculture. *Applied and Environmental Microbiology*.

[2] Passera A., Venturini G., Battelli G. (2017). Competition assays revealed Paenibacillus pasadenensis strain R16 as a novel antifungal agent. *Microbiological Research*.

[3] Wu L., Shang H., Wang Q., Gu H., Liu G., Yang S. (2016). Isolation and characterization of antagonistic endophytes from Dendrobium candidum Wall ex Lindl., and the biofertilizing potential of a novel Pseudomonas saponiphila strain. *Applied Soil Ecology*.

[4] Tan H. M., Cao L. X., He Z. F., Su G. J., Lin B., Zhou S. N. (2006). Isolation of endophytic actinomycetes from different cultivars of tomato and their activities against Ralstonia solanacearum in vitro. *World Journal of Microbiology and Biotechnology*.

[5] Yang W., Xu Q., Liu H.-X. (2012). Evaluation of biological control agents against Ralstonia wilt on ginger. *Biological Control*.

[6] Farina R., Beneduzi A., Ambrosini A. (2012). Diversity of plant growth-promoting rhizobacteria communities associated with the stages of canola growth. *Applied Soil Ecology*.

[7] Yaoyao E., Yuan J., Yang F. (2017). PGPR strain Paenibacillus polymyxa SQR-21 potentially benefits watermelon growth by re-shaping root protein expression. *AMB Express*.

[8] Molinatto G., Puopolo G., Sonego P. (2016). Complete genome sequence of Bacillus amyloliquefaciens subsp. plantarum S499, a rhizobacterium that triggers plant defences and inhibits fungal phytopathogens. *Journal of Biotechnology*.

[9] Pliego C., De Weert S., Lamers G. (2008). Two similar enhanced root-colonizing Pseudomonas strains differ largely in their colonization strategies of avocado roots and Rosellinia necatrix hyphae. *Environmental Microbiology*.

[10] Fließbach A., Winkler M., Lutz M. P., Oberholzer H.-R., Mäder P. (2009). Soil amendment with pseudomonas fluorescens CHA0: Lasting effects on soil biological properties in soils low in microbial biomass and activity. *Microbial Ecology*.

[11] Compant S., Duffy B., Nowak J., Clément C., Barka E. A. (2005). Use of plant growth-promoting bacteria for biocontrol of plant diseases: principles, mechanisms of action, and future prospects. *Applied and Environmental Microbiology*.

[12] Ahmad F., Ahmad I., Khan M. S. (2008). Screening of free-living rhizospheric bacteria for their multiple plant growth promoting activities. *Microbiological Research*.

[13] Alabouvette C., Olivain C., Migheli Q., Steinberg C. (2009). Microbiological control of soil-borne phytopathogenic fungi with special emphasis on wilt-inducing Fusarium oxysporum. *New Phytologist*.

[14] Von Der Weid I., Paiva E., Nóbrega A., Dirk Van Elsas J., Seldin L. (2000). Diversity of Paenibacillus polymyxa strains isolated from the rhizosphere of maize planted in Cerrado soil. *Research in Microbiology*.

[15] Gopalakrishnan S., Humayun P., Kiran B. K. (2011). Evaluation of bacteria isolated from rice rhizosphere for biological control of charcoal rot of sorghum caused by *Macrophomina phaseolina* (Tassi) Goid. *World Journal of Microbiology and Biotechnology*.

[16] Watanabe K., Kodama Y., Harayama S. (2001). Design and evaluation of PCR primers to amplify bacterial 16S ribosomal DNA fragments used for community fingerprinting. *Journal of Microbiological Methods*.

[17] Gal-Hemed I., Atanasova L., Komon-Zelazowska M., Druzhinina I. S., Viterbo A., Yarden O. (2011). Marine isolates of Trichoderma spp. As potential halotolerant agents of biological control for arid-zone agriculture. *Applied and Environmental Microbiology*.

[18] Wang X., Wang C., Li Q. (2018). Isolation and characterization of antagonistic bacteria with the potential for biocontrol of soil-borne wheat diseases. *Journal of Applied Microbiology*.

[19] Chuankun X., Minghe M., Leming Z., Keqin Z. (2004). Soil volatile fungistasis and volatile fungistatic compounds. *Soil Biology & Biochemistry*.

[20] Gull M. (2012). Characterization of siderophore producing bacterial strain Pseudomonas fluorescens Mst 8.2 as plant growth promoting and biocontrol agent in wheat. *African Journal of Microbiology Research*.

[21] Schwyn B., Neilands J. B. (1987). Universal chemical assay for the detection and determination of siderophores. *Analytical Biochemistry*.

[22] Abiala M. A., Odebode A. C., Hsu S. F., Blackwood C. B. (2015). Phytobeneficial properties of bacteria isolated from the rhizosphere of maize in southwestern Nigerian soils. *Applied and Environmental Microbiology*.

[23] Lü C., Huang B. L. (2010). Isolation and characterization of azotobacteriafrom pine rhizosphere. *African Journal of Microbiology Research*.

[24] Hassan W., Hussain M., Bashir S., Shah A. N., Bano R., David J. (2015). ACC-deaminase and/or nitrogen fixing rhizobacteria and growth of wheat (Triticum Aestivum L.). *Soil Science & Plant Nutrition*.

[25] Collavino M. M., Sansberro P. A., Mroginski L. A., Aguilar O. M. (2010). Comparison of in vitro solubilization activity of diverse phosphate-solubilizing bacteria native to acid soil and their ability to promote Phaseolus vulgaris growth. *Biology and Fertility of Soils*.

[26] Zheng J., Xiao X., Zhang Q., Mao L., Yu M., Xu J. (2015). The placental microbiome varies in association with low birth weight in full-term neonates. *Nutrients*.

[27] Ryu C. M., Farag M. A., Hu C. H. (2003). Bacterial volatiles promote growth in Arabidopsis. *Proceedings of the National Academy of Sciences of the United States of America*.

[28] Gu Y.-Q., Mo M.-H., Zhou J.-P., Zou C.-S., Zhang K.-Q. (2007). Evaluation and identification of potential organic nematicidal volatiles from soil bacteria. *Soil Biology & Biochemistry*.

[29] Aonuma S., Watanabe A., Onuma K., Sasaki M., Oizumi K., Konno K. (2011). Comparative studies of antibacterial effect of some antibiotics and ginger (Zingibe officinale) on two pathogenic bacteria. *Journal of Microbiology and Antimicrobials*.

[30] Fernando W. G. D., Ramarathnam R., Krishnamoorthy A. S., Savchuk S. C. (2005). Identification and use of potential bacterial organic antifungal volatiles in biocontrol. *Soil Biology & Biochemistry*.

[31] Williamson B., Tudzynski B., Tudzynski P., Van Kan J. A. L. (2007). Botrytis cinerea: the cause of grey mould disease. *Molecular Plant Pathology*.

[32] Corlett M., MacLatchy L. (1996). Alternaria brassicae. *Canadian Journal of Plant Pathology*.

[33] Yuan J., Raza W., Shen Q., Huang Q. (2012). Antifungal activity of bacillus amyloliquefaciens NJN-6 volatile compounds against Fusarium oxysporum f. sp. cubense. *Applied and Environmental Microbiology*.

[34] Goswami R. S., Kistler H. C. (2004). Heading for disaster: Fusarium graminearum on cereal crops. *Molecular Plant Pathology*.

[35] Ogoshi A. (1987). Ecology and pathogenicity of anastomosis and intraspecific groups of rhizoctonia solani kuhn. *Annual Review of Phytopathology*.

[36] Raaijmakers J. M., Paulitz T. C., Steinberg C., Alabouvette C., Moënne-Loccoz Y. (2009). The rhizosphere: a playground and battlefield for soilborne pathogens and beneficial microorganisms. *Plant and Soil*.

[37] Holl F. B., Chanway C. P., Turkington R., Radley R. A. (1988). Response of crested wheatgrass (Agropyron cristatum L.), perennial ryegrass (Lolium perenne and white clover (Trifolium repens L.) to inoculation with Bacillus polymyxa. *Soil Biology & Biochemistry*.

[38] Timmusk S., Wagner E. G. H. (1999). The plant-growth-promoting rhizobacterium Paenibacillus polymyxa induces changes in Arabidopsis thaliana gene expression: A possible connection between biotic and abiotic stress responses. *Molecular Plant-Microbe Interactions*.

[39] Kobayashi D. Y., Reedy R. M., Bick J., Oudemans P. V. (2002). Characterization of a chitinase gene from Stenotrophomonas maltophilia strain 34S1 and its involvement in biological control. *Applied and Environmental Microbiology*.

[40] Kilic-Ekici O., Yuen G. Y. (2003). Induced resistance as a mechanism of biological control by Lysobacter enzymogenes strain C3. *Journal of Phytopathology*.

[41] Fernando W. G. D., Nakkeeran S., Zhang Y. (2005). *Biosynthesis of Antibiotics by PGPR and Its Relation in Biocontrol of Plant Diseases*.

[42] Ryu C. M., Farag M. A., Hu C. H. (2013). Bacterial volatiles promote growth in *Arabidopsis*. *Proceedings of the National Acadamy of Sciences of the United States of America*.

[43] Fiddaman P. J., Rossall S. (1994). Effect of substrate on the production of antifungal volatiles from Bacillus subtilis. *Journal of Applied Bacteriology*.

[44] Mckee N. D., Robinson P. M. (1988). Production of volatile inhibitors of germination and hyphal extension by geotrichum candidum. *Transactions of the British Mycological Society*.

[45] Dilantha Fernando W. G., Linderman R. G. (2012). Inhibition of *Phytophthora vignae* and stem and root rot of cowpea by soil bacteria. *Biological Agriculture & Horticulture*.

[46] Kaufman P. B., Song I. (1995). Hormones and the Orientation of Growth. *Plant Hormones and Their Role in Plant Growth and Development*.

[47] Urrea R., Cabezas L., Sierra R., Cárdenas M., Restrepo S., Jiménez P. (2011). Selection of antagonistic bacteria isolated from the Physalis peruviana rhizosphere against Fusarium oxysporum. *Journal of Applied Microbiology*.

[48] Khan N. I., Schisler D. A., Boehm M. J., Slininger P. J., Bothast R. J. (2001). Selection and evaluation of microorganisms for biocontrol of Fusarium head blight of wheat incited by Gibberella zeae. *Plant Disease*.

[49] Kumar A., Prakash A., Johri B. N. (2011). Bacillus as PGPR in Crop Ecosystem. *Chapter 2*.

[50] Sun J., Zhang Q., Zhou J., Wei Q. (2014). Pyrosequencing technology reveals the impact of different manure doses on the bacterial community in apple rhizosphere soil. *Applied Soil Ecology*.

[51] Dun-Dun J. I., Qiu H. D., Xie H. (2011). The improvement of N-ammonia degradation of photosynthetic bacteria by protoplast fusion. *Journal of Dalian Ocean University*.

[52] Lauber C. L., Hamady M., Knight R., Fierer N. (2009). Pyrosequencing-based assessment of soil pH as a predictor of soil bacterial community structure at the continental scale. *Applied and Environmental Microbiology*.

[53] Franke-Whittle I. H., Manici L. M., Insam H., Stres B. (2015). Rhizosphere bacteria and fungi associated with plant growth in soils of three replanted apple orchards. *Plant and Soil*.

[54] Horino O. (2002). Studies on resistance mechanisms of rice against bacterial blight caused by xanthomonas oryzae pv. oryzae. *Journal of General Plant Pathology*.

[55] Vicente J. G., Holub E. B. (2013). Xanthomonas campestris pv. Campestris (cause of black rot of crucifers) in the genomic era is still a worldwide threat to brassica crops. *Molecular Plant Pathology*.

[56] Ando S., Ito T., Kanno T. (2014). Impact of organic crop management on suppression of bacterial seedling diseases in rice. *Organic Agriculture*.

[57] Kavia F. (2010). *B. Xanthomonas, Impact of Banana Bacterial Wilt Disease in Tanzania*.

[58] Qiao J., Yu X., Liang X., Liu Y., Borriss R., Liu Y. (2017). Addition of plant-growth-promoting Bacillus subtilis PTS-394 on tomato rhizosphere has no durable impact on composition of root microbiome. *BMC Microbiology*.

