# Supplementary material for: Isolation and Characterization of Antagonistic Bacteria Paenibacillus jamilae HS-26 and Their Effects on Plant Growth
Source: Biomed Res Int. 2019 Mar 27;2019:3638926. doi: 10.1155/2019/3638926 (PMC6457365; doi:10.1155/2019/3638926)
Supplement: Supplementary Materials — Figure S1: Effects of HS-26 on cucumber seed germination and growth. Seeds germination assay. (a) Seeds coated with HS-26 strain cells; (b) seeds uncoated with HS-26 strain cells as blank control; (c) growth promotion of cucumber seedlings by Paenibacillus jamilae HS-26 compared with the untreated control. Figure S2: bacterial and fungal rarefaction curves and Shannon curves depicting the effect of strain HS-26 and control treatments on the number of OTUs. (a) Rarefaction curves of bacteria from HS-26 and control treatments; (b) Shannon curves of bacteria from HS-26 and control treatments; (c) rarefaction curves of fungi from HS-26 and control treatments; (d) Shannon curves of fungi from HS-26 and control treatments. Figure S3: the relative abundance (%) of all bacteria and fungi on the phylum level in the rhizosphere soil of HS-26 and control treatments. (a) The relative abundance of all detected bacterial phyla in control group; (b) the relative abundance of all detected bacterial phyla in HS-26 treatment; (c) the relative abundance of all detected fungal phyla in control group; (d) the relative abundance of all detected fungal phyla in HS-26 treatment. Table S1: HS-26 effects on the germination rate and growth of cucumber. Table S2: percent of pathogenic fungi abundance in HS-26 treatment and control groups. [file 3638926.f1.docx]

**Supplementary material**

**Isolation and characterization of antagonistic bacteria****l strain *Paenibacillus jamilae* HS-26 and its effects on plant growth**

***Biomed Research International***

Xiaohui Wang^1^, Qian Li^2^_,_ Junkang Sui^1^, Jiamiao Zhang^2^, Zhaoyang Liu^2^, Jianfeng Du^2^, Ruiping Xu^2^, Yanyan Zhou^2^, Xunli Liu^2b^*

^1^ College of Life Science, Shandong Agriculture University, No. 61, Daizong Street, Taian, Shandong 271018, China

^2^ College of Forestry, Shandong Agricultural Universities, No. 61, Daizong Street, Taian, Shandong 271018, China

* Author for correspondence, E-mail: xunliliu@163.com

Tel: 0086 0538 8249131

Fax: 0086 0538 8249164

**Figure S1**


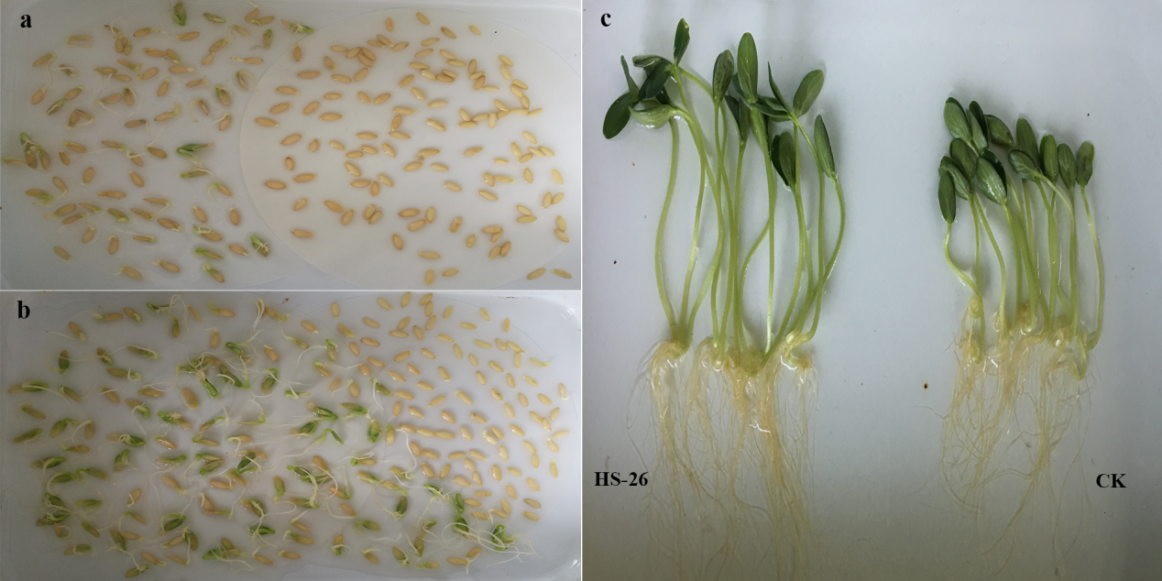


Fig.S1 Effects of HS-26 on cucumber seed germination and growth. Seeds germination assay. (a): seeds coated with HS-26 strain cells; (b) seeds un-coated with HS-26 strain cells as blank control; (c) Growth promotion of cucumber seedlings by *Paenibacillus jamilae* HS-26 compared with the untreated control.

**Figure S2**


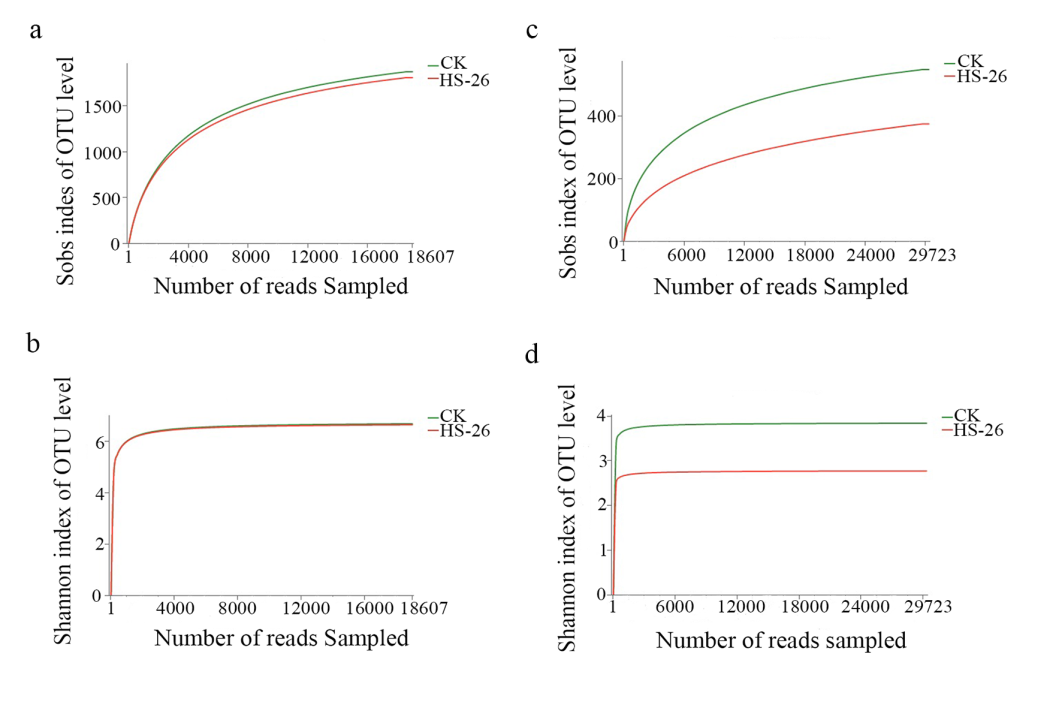


Fig.S2 Bacterial and fungal rarefaction curves and Shannon curves depicting the effect of strain HS-26 and control treatments on the number of OTUs. a: Rarefaction curves of bacteria from HS-26 and control treatments; b: Shannon curves of bacteria from HS-26 and control treatments; c: Rarefaction curves of fungi from HS-26 and control treatments; d: Shannon curves of fungi from HS-26 and control treatments

**Figure S3**

**
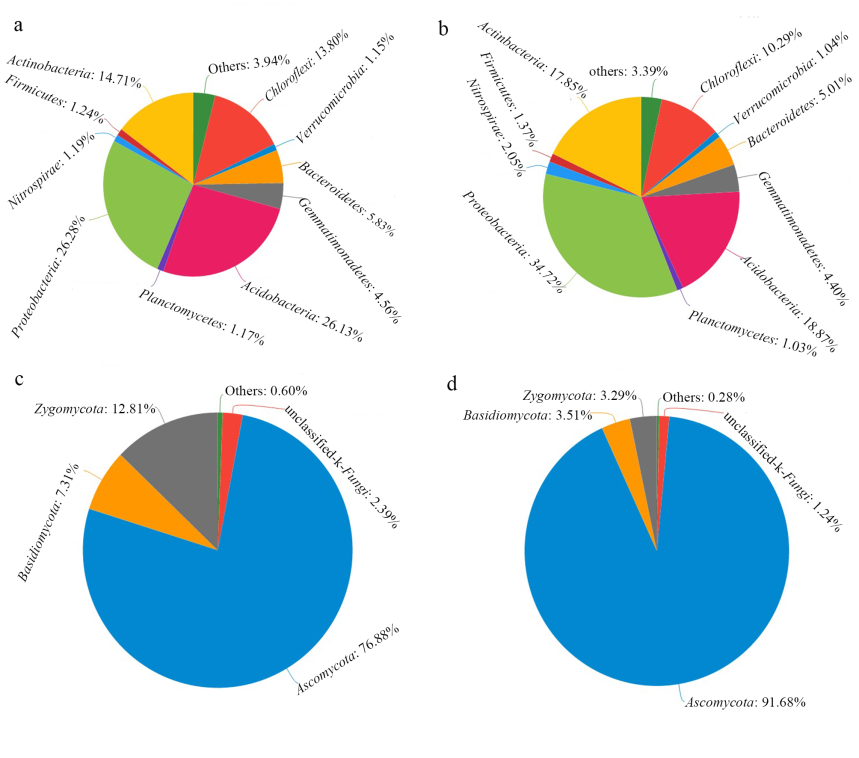
**

Fig.S3. The relative abundance (%) of all bacteria and fungi on the phylum level in the rhizosphere soil of HS-26 and control treatments. a: The relative abundance of all detected bacterial phyla in control group; b: the relative abundance of all detected bacterial phyla in HS-26 treatment; c: The relative abundance of all detected fungal phyla in control group; d: The relative abundance of all detected fungal phyla in HS-26 treatment.

**Tables**

Table S1 HS-26 effects on the germination rate and growth of cucumber

| Treatment | Germination rate (%) | Root length (cm) | Shoot height (cm)^b^ | Fresh weight (g) | Dry weight (g) |
| --- | --- | --- | --- | --- | --- |
| CK | 66.0 ± 3.6b | 7.65±1.34b | 4.16 ±0.2b | 1.28±0.10b | 0.48 ±0.01b |
| HS-26 | 75.2 ± 2.0a | 9.12±0.20a | 5.43±0.0.79a | 1.43 ±0.08a | 0.50 ±0.00a |

Note: Values represent mean values ± SD (n = 30). The same letters in the same columns indicate no significant differences (*p* >0.05). HS-26 refers to cucumber seedlings treated with HS-26 cell suspension. CK refers to cucumber seedlings treated with an equal volume of sterile water.

Table S2 Percent of pathogenic fungi abundance in HS-26 treatment and control groups

| Treatment | *Fusarium* | *Gibberella* | *Monographella* | *Volutella* | *Bipolaris* | *Idriella* |
| --- | --- | --- | --- | --- | --- | --- |
| CK | 5.83%±0.01a | 7.45%±0.01a | 1.13%±0.00a | 0.72%±0.00a | 0.02%±0.00a | 0.02%±0.00a |
| HS-26 | 1.80%±0.00b | 2.57%±0.01b | 0.35%±0.00b | 0.13%±0.00b | 0 | 0 |

Notes: Values are the means ± SD (n = 3). Means sharing a common letter within the same column are not significantly different at P < 0.05.
